# Connexin43 Containing Gap Junction Channels Facilitate HIV Bystander Toxicity: Implications in NeuroHIV

**DOI:** 10.3389/fnmol.2017.00404

**Published:** 2017-12-05

**Authors:** Shaily Malik, Martin Theis, Eliseo A. Eugenin

**Affiliations:** ^1^Public Health Research Institute (PHRI), Newark, NJ, United States; ^2^Department of Microbiology, Biochemistry, and Molecular Genetics, Rutgers New Jersey Medical School, Rutgers, The State University of New Jersey, Newark, NJ, United States; ^3^Institute of Cellular Neurosciences, University of Bonn, Bonn, Germany

**Keywords:** NeuroAIDS, HIV, connexin43, astrocyte, apoptosis

## Abstract

Human immunodeficiency virus-1 (HIV-1) infection compromises the central nervous system (CNS) in a significant number of infected individuals, resulting in neurological dysfunction that ranges from minor cognitive deficits to frank dementia. While macrophages/microglia are the predominant CNS cells infected by HIV, our laboratory and others have shown that HIV-infected astrocytes, although present in relatively low numbers with minimal to undetectable viral replication, play key role in NeuroAIDS pathogenesis. Our laboratory has identified that HIV “hijacks” connexin (Cx) containing channels, such as gap junctions (GJs) and hemichannels (HCs), to spread toxicity and apoptosis to uninfected cells even in the absence of active viral replication. In this study, using a murine model with an astrocyte-directed deletion of Cx43 gene (hGFAP-cre Cx43^fl/fl^) and control Cx43^fl/fl^ mice, we examined whether few HIV-infected human astrocytoma cells (U87-CD4-CCR5), microinjected into the mouse cortex, can spread toxicity and apoptosis through GJ-mediated mechanisms, into the mouse cells, which are resistant to HIV infection. In the control Cx43^fl/fl^ mice, microinjection of HIV-infected U87-CD4-CCR5 cells led to apoptosis in 84.28 ± 6.38% of mouse brain cells around the site of microinjection, whereas hGFAP-cre Cx43^fl/fl^ mice exhibited minimal apoptosis (2.78 ± 1.55%). However, simultaneous injection of GJ blocker, 18α-glycyrrhetinic acid, and Cx43 blocking peptide along with microinjection of HIV-infected cells prevented apoptosis in Cx43^fl/fl^ mice, demonstrating the Cx43 is essential for HIV-induced bystander toxicity. In conclusion, our findings demonstrate that Cx43 expression, and formation of GJs is essential for bystander apoptosis during HIV infection. These findings reveal novel potential therapeutic targets to reduce astrocyte-mediated bystander toxicity in HIV-infected individuals because despite low to undetectable viral replication in the CNS, Cx channels hijacked by HIV amplify viral neuropathogenesis.

## Introduction

As reported by UNAIDS in 2015, approximately 36 million people are infected with human immunodeficiency virus-1 (HIV-1) worldwide (UNAIDS, 2016). In addition to immune compromise, HIV infection causes a wide spectrum of HIV-associated neurocognitive disorders (HAND) which range from mild cognitive disease to dementia, even in the context of successful anti-retroviral therapy (ART) (Anthony et al., 2005; Antinori et al., 2007; Heaton et al., 2010). HIV infection of the central nervous system (CNS) occurs within weeks of systemic infection, when circulating HIV-infected monocytes/macrophages enter the brain, and infect CNS-resident microglia, macrophages, and a small population of astrocytes (Wiley et al., 1999; Churchill et al., 2006). In the CNS, HIV-infected cells release a plethora of host and viral molecules (e.g., chemokines/cytokines and viral proteins, respectively), which promote neuroinflammation, even during ART when HIV replication is undetectable (Clifford, 2017). Interestingly, upon interruption of the ART, the virus re-emerges, suggesting existence of long-lasting viral reservoirs in HIV-infected individuals (Chun et al., 1999). Furthermore, despite peripheral control of HIV replication by ART, 50–60% of HIV-infected individuals have some degree of cognitive disease (Heaton et al., 2010), suggesting that HIV uses unknown replication-independent mechanisms to amplify damage within the CNS. Work from our laboratory has demonstrated that one of the mechanisms of CNS damage is elicited by HIV-infected astrocytes, which, while representing a small fraction of all astrocytes, act as a source of toxic, pro-apoptotic signals that spread to neighboring uninfected cells via connexin43 (Cx43) containing channels, gap junctions (GJ) and unopposed hemichannels (uHC), causing “bystander apoptosis” despite absence of viral replication (Eugenin and Berman, 2007, 2013; Eugenin et al., 2011; Orellana et al., 2014).

Gap junctions connect the cytoplasm of two adjoining cells enabling direct exchange of cytoplasmic products between the connected cells. Each GJ channel is formed from the docking of two hemichannels (HCs), and each HC comprises of a hexameric assembly of connexin (Cx) protein subunits (Bennett et al., 2003). Recently, our laboratory has identified that uHCs open under HIV-infected conditions enabling the release of intracellular factors into the extracellular milieu (Orellana et al., 2014). Both types of channels, GJs and uHCs, can pass molecules up to 1.2 kDa in size, including second messengers, ions, ATP, prostaglandins, small peptides, and RNA (Saez et al., 2003). Previously, we have demonstrated that HIV-infected astrocytes “hijack” GJs and uHCs to spread toxic, pro-apoptotic, and pro-inflammatory molecules, such as inositol triphosphate (IP_3_), Ca^2+^, glutamate, ATP, and prostaglandin E2 (PGE_2_), to vast areas of the CNS, into uninfected cells eliciting neuronal and glial compromise (Eugenin and Berman, 2007, 2013; Eugenin et al., 2011). Interestingly, in contrast to the uninfected cells, HIV-infected astrocytes were resistant to pro-apoptotic signals, and exhibited extended survival due to reduced mitochondrial function, IP_3_ receptor sensitivity, calcium response, and apoptosome formation (Eugenin and Berman, 2013). Importantly, all of these mechanisms were independent of HIV replication, and occur even in the presence of successful ART. We have also demonstrated that most of these mechanisms operate *in vivo* (i.e., in human and monkey brain tissue samples), underscoring the importance of glial cells and Cx43 containing channels in the pathogenesis of NeuroAIDS (Berman et al., 2016).

In the present study, we microinjected HIV-infected U87-CD4-CCR5 cells into the cortex of Cx43-expressing (Cx43^fl/fl^) and Cx43-deficient (hGFAP-cre Cx43^fl/fl^) mice, and assessed bystander apoptosis in the mouse cells. We demonstrate that Cx43 in astrocytes is essential for amplifying bystander apoptosis from few HIV-infected astrocytes to neighboring mouse cells which do not support viral replication. The mechanism of bystander apoptosis was dependent not only on Cx43 but also on IP_3_ receptor activation and intracellular calcium. Thus, we propose that HIV “hijacks” Cx43 containing channels even in the absence of HIV replication to amplify damage within the CNS.

## Materials and Methods

### Materials

U87-CD4-CCR5 cells, HIV_ADA_, and HIV-1 p24 antibody were obtained from NIH AIDS Reagent program (Germantown, MD, United States). Dulbecco’s modified Eagle’s medium (DMEM), fetal bovine serum (FBS), penicillin/streptomycin, trypsin-EDTA, secondary antibody conjugated to FITC, and prolong gold anti-fade reagent with DAPI were purchased from Thermo Fisher Scientific (Waltham, MA, United States). The *in situ* cell death detection kit, TUNEL was purchased from Roche (Mannheim, Germany). BAPTA-AM, 18α-glycyrrhetinic acid (AGA), and isoflurane were procured from Sigma–Aldrich (St. Louis, MO, United States). Xestospongin C (XeC) was purchased from Tocris (Bristol, United Kingdom). Cx43 blocking peptide and scrambled (Scr) peptide were obtained from PeproTech (Rocky Hill, NJ, United States).

### Methods

#### Cx43 Conditional Knockout Mice

**FIGURE 1:**
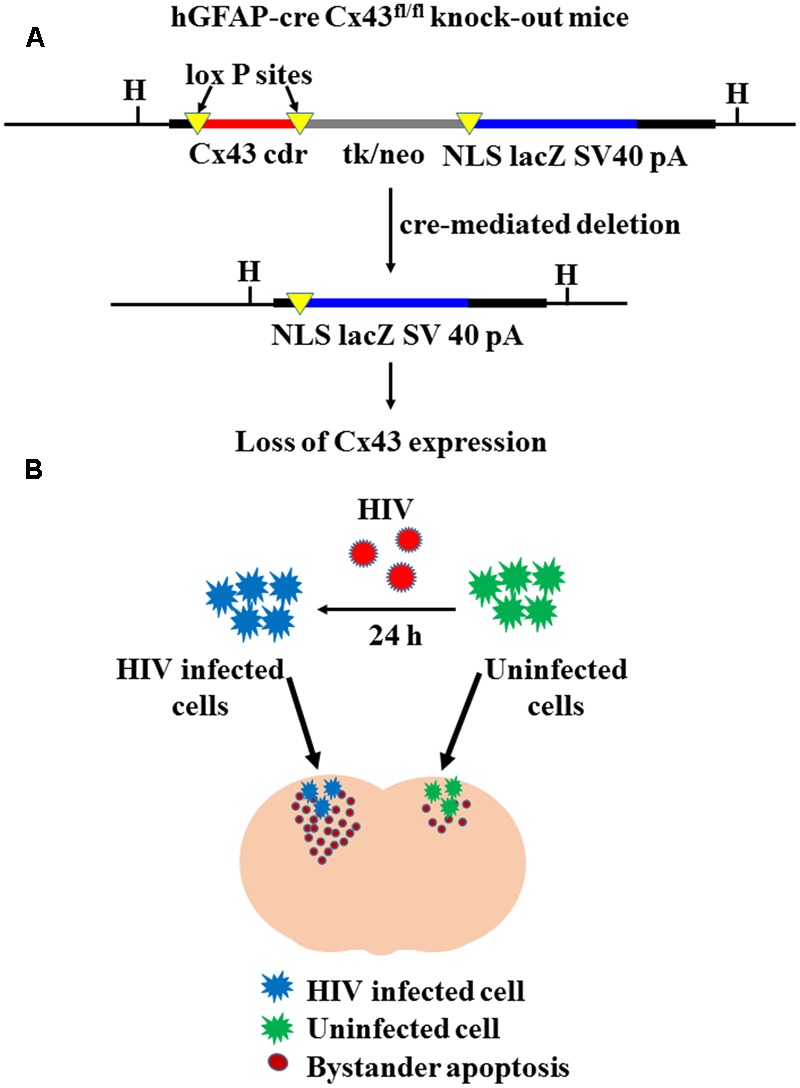
Cre-mediated deletion of Cx43 expression. **(A)** Represents the concept of cre-mediated deletion of floxed DNA at the Cx43 locus, leading to expression of lacZ in the cells that display Cx43 gene activity. Since *cre* transgene is under the human GFAP promoter (hGFAP-cre), Cx43 is specifically deleted in astrocytes. Cx43 cdr: Cx43 coding region; tk/neo: herpes simplex virus thymidine-kinase/neomycin phosphotransferase; NLS lacZ: lacZ reporter gene with nuclear localization signal; SV40 pA: SV40 polyadenylation signal; H: HindIII restriction site [Adapted from Theis et al. (2003)]. **(B)** Represents experimental strategy of microinjecting HIV-infected/uninfected human U87-CD4-CCR5 cells in Cx43^fl/fl^ and hGFAP-cre Cx43^fl/fl^ mice. Human U87-CD4-CCR5 astrocytoma cells were infected with HIV_ADA_ (50 ng/ml) for 24 h, and then microinjected into cortices of Cx43^fl/fl^ and hGFAP-cre Cx43^fl/fl^ mice. After 48 h of microinjection, extent of bystander toxicity was analyzed in the mouse cortex by detecting apoptosis with TUNEL assay. In the contra-lateral hemisphere, mice were injected with uninfected U87-CD4-CCR5 cells, which served as control for microinjection of HIV-infected cells.

Mice with astrocyte-specific deletion of Cx43 were generated by interbreeding of Cx43^fl^ mice (Theis et al., 2001), in which the Cx43 coding region is flanked by loxP sites, with mice carrying a *cre* re-combinase transgene under the human glial fibrillary acidic protein (GFAP) promoter, hGFAP-cre (Zhuo et al., 2001) (**Figure 1A**). In astrocyte cultures from hGFAP-cre Cx43^fl/fl^ mice, at least 90% of the cells displayed loss of Cx43 expression after 4 weeks in culture (Theis et al., 2003). Similar results of loss of Cx43 expression (approximately 90%) were observed for our animals using Western blotting (data not shown). For the present study, we used five adult male hGFAP-cre Cx43^fl/fl^ mice, and as controls, four adult male Cx43^fl/fl^ mice.

#### HIV Infection of U87-CD4-CCR5 Cells

Human astrocytoma cells transfected with CD4 and CCR5, U87-CD4-CCR5, were obtained from NIH AIDS Reagent Program (Germantown, MD, United States). CD4 and CCR5 transfection enables the U87 cells to achieve 90–95% infection with HIV, and is widely used to study HIV infection in astrocytes (Bjorndal et al., 1997; Orellana et al., 2014). U87-CD4-CCR5 cells were grown in DMEM supplemented with 10% FBS and 1% penicillin/streptomycin. Prior to microinjection, U87-CD4-CCR5 cells were infected with HIV_ADA_ (50 ng/ml) for 24 h (**Figure 1B**). The cells were washed once with the media to remove any free virus. The cells were then re-suspended in phosphate buffered saline (PBS) at a density of 3,000 cells/0.5 μl for microinjection.

#### Microinjection of U87-CD4-CCR5 Cells in Mouse Cortex

For microinjection, four adult male Cx43^fl/fl^ mice and five adult male hGFAP-cre Cx43^fl/fl^ mice, 6 weeks of age were used in the study. The mice were anesthetized by isoflurane inhalation for the procedure (nose cone providing continuous 1.5% isoflurane). The microinjection of U87-CD4-CCR5 cells was performed as described in approved IACUC protocol 12016D015 (Rutgers University, NJ, United States). Once anesthetized, the top of the animal’s head was shaved, and sterilized with a betadine pad. A 0.5 cm incision was made, and the skin was folded back to expose the top of the cranium. A 0.01 cm hole was made into the top of the cranium using a small drill exposing the mouse brain. In this opening, a Hamilton Neuros syringe was used to inject 3,000 HIV-infected U87-CD4-CCR5 astrocytoma cells in total a volume of 0.5 μl, at four different sites in the gray matter of the somatosensory cortex (S1). The syringe needle had a fixed length (0.1 cm) to ensure that the injection is made to the cortex. After the injection, the skin was sutured using tissue adhesive. As a control, the same animal was injected with uninfected U87-CD4-CCR5 cells in a similar manner in the contralateral hemisphere, to negate the effects of host immune response on the results. The mice were kept under continuous observation after the procedure. For experiments with chemical and peptide blockers, the following concentrations were used: 18α-glycyrrhetinic acid (AGA): 35 μM; Cx43 blocking peptide: 100 μM; Scr peptide: 100 μM; Xestospongin C: 10 μM; BAPTA-AM: 5 μM. As per the experiment, either inhibitors or peptides were added to the cell mix (3,000 cells/0.5 μl) prior to microinjection. All these blockers were tested in different cell lines and models to assure specificity, concentration and toxicity (Eugenin and Berman, 2007, 2013). As a control, the same animal was injected with either HIV-infected or uninfected U87-CD4-CCR5 cells without the inhibitor/peptide in the contralateral hemisphere. After 48 h of microinjection, the mice were sacrificed by cervical dislocation and brains were harvested. We concluded our experiments 48 h post-microinjection because bystander apoptosis was evident at that time, and to reduce pain and suffering of the animals.

#### Immunohistochemistry and Apoptosis Assay

Harvested mice brains were fixed in 4% paraformaldehyde for 24 h and then transferred to 30% sucrose solution at 4°C. After 48 h, the brains were sectioned into 10-μm-thick coronal sections. For each animal, 20–40 sections were analyzed to reconstruct the injected area and to quantify bystander killing. Apoptosis was detected by terminal deoxynucleotidyl transferase dUTP nick end labeling (TUNEL) assay as described previously (Eugenin and Berman, 2013). Briefly, tissue sections were incubated in TUNEL reaction mixture (Enzyme solution:Label solution 1:9) at 37°C for 1 h, washed three times with PBS, and proceeded for immunohistochemistry. The tissue sections were permeabilized with 0.01% Triton × 100, washed three times with PBS, and then incubated in blocking solution (50 μM EDTA, 1% fish gelatin, 1% bovine serum albumin, 2% human serum, and 2% horse serum) for 2 h at room temperature (RT). The samples were then incubated with HIV-1 p24 antibody (1:50), overnight at 4°C. Then, the tissue samples were washed several times with PBS at RT, and incubated with the appropriate secondary antibody conjugated to FITC for 2 h at RT. Following several washes with PBS, the tissue sections were mounted with prolong gold anti-fade reagent with DAPI. The tissue samples were examined by Leica SP2 confocal microscope (Leica, Germany), and analyzed by Adobe Photoshop, NIH ImageJ, and Nikon NIS Elements software. For calculating the percentage of apoptotic cells around the site of microinjection, a circular area of 200 μm radius from the site of microinjection was assessed for the presence of TUNEL-positive nuclei. Percentage of apoptotic cells was calculated by counting the total number of TUNEL-positive nuclei versus the total number of nuclei stained with DAPI in the circular area of 200 μm radius from the site of microinjection.

#### Statistical Analysis

All data are presented as mean ± standard deviation (SD). Each animal was microinjected at four sites, and in the figures, one dot represents one microinjection. All statistical analysis have been performed using one-way ANOVA. The *p*-values < 0.05 were considered significant.

## Results

### Microinjection of HIV-Infected Human Astrocytoma Cells into the Brain of Cx43 Expressing Mouse Induces Bystander Apoptosis in Mouse Cells

To examine bystander apoptosis in the absence of HIV infection, we microinjected 3000 U87-CD4-CCR5 cells into the cortex of mice containing astrocyte-specific deletion of Cx43, hGFAP-cre Cx43^fl/fl^, and control, Cx43^fl/fl^ mice (with normal expression of Cx43) (**Figure 1B**). The U87-CD4-CCR5 cells were infected with HIV_ADA_ (50 ng/ml) for 24 h, and based on HIV-p24 immunostaining, approximately 90–95% cells were infected after the first round of replication (data not shown). Specifically, 3000 HIV-infected U87-CD4-CCR5 cells were microinjected at four sites in the somatosensory cortex (S1) of hGFAP-cre Cx43^fl/fl^ and Cx43^fl/fl^ mice. As a control, equal number of uninfected U87-CD4-CCR5 cells was microinjected into the contralateral hemisphere of the same animal. After 48 h of microinjection, the animals were sacrificed, and the brains were sectioned and analyzed for apoptosis.

**FIGURE 2:**
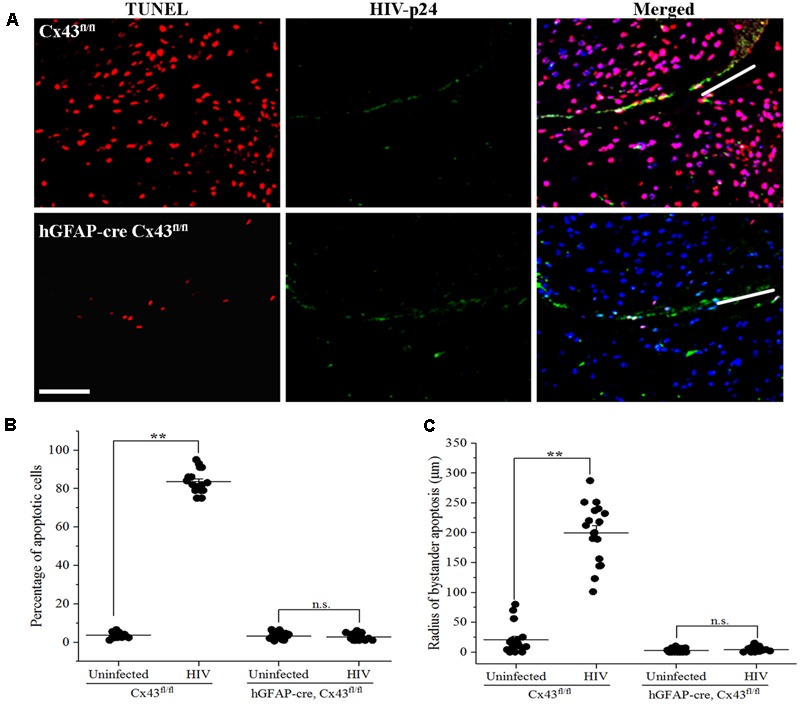
Microinjection of HIV-infected cells induces bystander apoptosis in mice cortex. Human U87-CD4-CCR5 cells infected with HIV_ADA_ (50 ng/ml) were microinjected into the cortices of Cx43^fl/fl^ as well as hGFAP-cre Cx43^fl/fl^ mice, and after 48 h, animals were sacrificed and brain sections were analyzed for apoptosis by TUNEL assay. **(A)** Representative images showing apoptosis (TUNEL, red) upon microinjection of HIV-infected astrocytes in Cx43^fl/fl^ as well as hGFAP-cre Cx43^fl/fl^ mice. HIV-p24 has been labeled with FITC (green), and cell nuclei with DAPI (blue). White lines in the merged images show the direction of microinjection. Scale bar denotes 75 μm. **(B)** Graphical representation of the extent of bystander apoptosis observed in a circular area of 200 μm around the site of microinjection in Cx43^fl/fl^ and hGFAP-cre Cx43^fl/fl^ mice. Microinjection of HIV-infected cells led to significant apoptosis in Cx43^fl/fl^ mice as GJs were formed between the microinjected human cells and murine host cells, which resulted in the diffusion of apoptotic factors generated in a few HIV-infected cells to host cells leading to their apoptosis. However, hGFAP-cre Cx43^fl/fl^ mice displayed minimal apoptosis even after microinjection of HIV-infected cells, possibly due to the absence of Cx43 GJs and HCs in these animals. **(C)** Represents the radius of bystander apoptosis which is the maximum distance of apoptotic cells from the site of microinjection of either HIV-infected or uninfected cells. The Cx43^fl/fl^ mice where HIV-infected cells were microinjected exhibit significantly larger radius of bystander apoptosis as compared to Cx43^fl/fl^ mice that received uninfected cells or hGFAP-cre Cx43^fl/fl^ mice that received either HIV-infected or uninfected cells. Each dot in the graphs **(B,C)** represents a microinjection. Each animal was microinjected at four sites; hence, in total, there were 16 microinjections for Cx43^fl/fl^ mice and 20 microinjections for hGFAP-cre Cx43^fl/fl^ mice. ^∗∗^*p* < 0.005; n.s. – not significant.

Microinjection of human HIV-infected astrocytoma cells into cortex of Cx43^fl/fl^ mice led to significant apoptosis, and approximately 84.28 ± 6.38% host cells were apoptotic in the region surrounding the site of microinjection [**Figures 2A**(Upper panel), **2B**]. In contrast, microinjection of HIV-infected human astrocytes into hGFAP-cre Cx43^fl/fl^ mice resulted in minimal apoptosis (2.78 ± 1.55%) around the site of microinjection [**Figures 2A**(Lower panel), **2B**]. Microinjection of uninfected human astrocytoma cells resulted in minimal apoptosis in Cx43^fl/fl^ (3.72 ± 1.44%) as well as in hGFAP-cre Cx43^fl/fl^ (3.23 ± 1.53%) mice at the site of microinjection (**Figure 2B** and **Table 1**). Microinjection of cell free virus (50 ng/ml) did not altered the levels of apoptosis (data not shown). Since HIV does not infect mouse cells as the viral envelope glycoprotein, gp120, does not engage mouse CD4 receptors (Browning et al., 1997), we propose that the microinjected human astrocytoma cells form GJs with resident mouse brain cells (Supplementary Figure S1 and Supplementary Data), and the cytoplasmic apoptotic factors generated in HIV-infected U87-CD4-CCR5 cells diffuse into uninfected host cells via Cx43 GJs and lead to their apoptosis.

**Table 1:** Bystander apoptosis in Cx43^fl/fl^ and hGFAP-cre Cx43^fl/fl^ mice upon microinjection of either uninfected or HIV-infected U87-CD4-CCR5 cells under various treatment conditions.

Treatment conditions	Cx43^fl/fl^ mice		hGFAP-cre Cx43^fl/fl^ mice	
	Uninfected cells	HIV-infected cells	Uninfected cells	HIV-infected cells
Control	3.72 ± 1.44%	84.28 ± 6.38%	3.23 ± 1.53%	2.78 ± 1.55%
	20.56 ± 23.63 μm	199.72 ± 49.52 μm	2.59 ± 2.86 μm	3.81 ± 3.57 μm
AGA	3.36 ± 1.26%	23.30 ± 7.51%	3.26 ± 1.56%	2.59 ± 1.15%
	20.72 ± 8.97 μm	51.61 ± 22.71 μm	4.07 ± 1.75 μm	5.93 ± 2.04 μm
Cx43 blocking peptide	3.53 ± 1.39%	19.22 ± 15.82%	3.21 ± 1.52%	3.67 ± 2.35%
	10.74 ± 1.62 μm	34.44 ± 6.54 μm	4.44 ± 1.74 μm	3.33 ± 3.11 μm
Scrambled peptide	3.92 ± 1.47%	80.56 ± 12.02%	3.31 ± 1.60%	5.07 ± 1.30%
	14.28 ± 5.82 μm	204.50 ± 32.72 μm	3.96 ± 2.24 μm	9.30 ± 3.73 μm
Xestospongin C	3.86 ± 1.48%	30.55 ± 16.15%	3.27 ± 1.48%	3.00 ± 1.41%
	11.56 ± 4.88 μm	51.36 ± 32.34 μm	2.41 ± 1.39 μm	2.22 ± 1.15 μm
BAPTA-AM	3.31 ± 1.50%	14.91 ± 6.54%	3.31 ± 1.45%	3.96 ± 1.83%
	10.72 ± 3.04 μm	23.28 ± 7.04 μm	5.71 ± 3.21 μm	4.53 ± 2.60 μm

*Data with “%” corresponds to the percentage of apoptotic cells in a circular area of 200 μm around the site of microinjection. Data with “μm” corresponds to the radius of bystander apoptosis which is the maximum distance of apoptotic cells from the site of microinjection.*

To quantify the degree of damage, we measured the radius of bystander apoptosis, as determined by the presence of apoptotic cells in a circular area from the site of microinjection of HIV-infected/uninfected cells. As shown in **Figure 2C**, Cx43^fl/fl^ mice expressing Cx43 GJ channels and HCs had amplification of apoptosis as upon microinjection of HIV-infected astrocytoma cells, TUNEL-positive cells were observed up to a distance of 199.72 ± 49.52 μm from the site of microinjection as opposed to 20.56 ± 23.63 μm when uninfected U87-CD4-CCR5 cells were microinjected (**Table 1**). However, microinjection of HIV-infected cells into hGFAP-cre Cx43^fl/fl^ mice resulted in minimal cell death (**Figure 2B**), and apoptotic cells were observed only up to a distance of 3.81 ± 3.57 μm from the site of microinjection, exhibiting much smaller radius of bystander apoptosis as compared to Cx43^fl/fl^ mice (**Figure 2C**). It is important to mention here that even microinjection of uninfected cells in hGFAP-cre Cx43^fl/fl^ mice had significantly smaller radius of toxicity (2.59 ± 2.86 μm) as compared to Cx43^fl/fl^ mice (20.56 ± 23.63 μm) (**Figure 2C**), which underscores the role of Cx43 channels in mediating the spread of toxic factors from damaged/pro-apoptotic cells to healthy cells during any pathology. Hence, we propose that the amplification of apoptosis was dependent on the microinjection of HIV-infected cells as well as on the presence of Cx43 GJ channels and HCs to propagate apoptotic stimuli from few HIV-infected cells to the uninfected cells.

### Functional GJs/HCs Are Required for Propagation of Apoptotic Factors from HIV-Infected Cells to Uninfected Cells

To further characterize the role of Cx43 containing GJs and HCs in mediating HIV-induced bystander apoptosis, inhibitor for GJ-mediated communication, 18α-glycyrrhetinic acid, AGA (35 μM), and Cx43 blocking peptide (100 μM), which blocks both Cx43 GJs and HCs, were used in the study. A Scr peptide was also used in the study as a negative control for Cx43 blocking peptide. At the time of microinjection, either AGA or Cx43 blocking peptide or Scr peptide was administered along with HIV-infected/uninfected U87-CD4-CCR5 cells into the cortices of hGFAP-cre Cx43^fl/fl^ and Cx43^fl/fl^ mice. After 48 h, the animals were sacrificed, and the brains were sectioned and analyzed for apoptosis.

**FIGURE 3:**
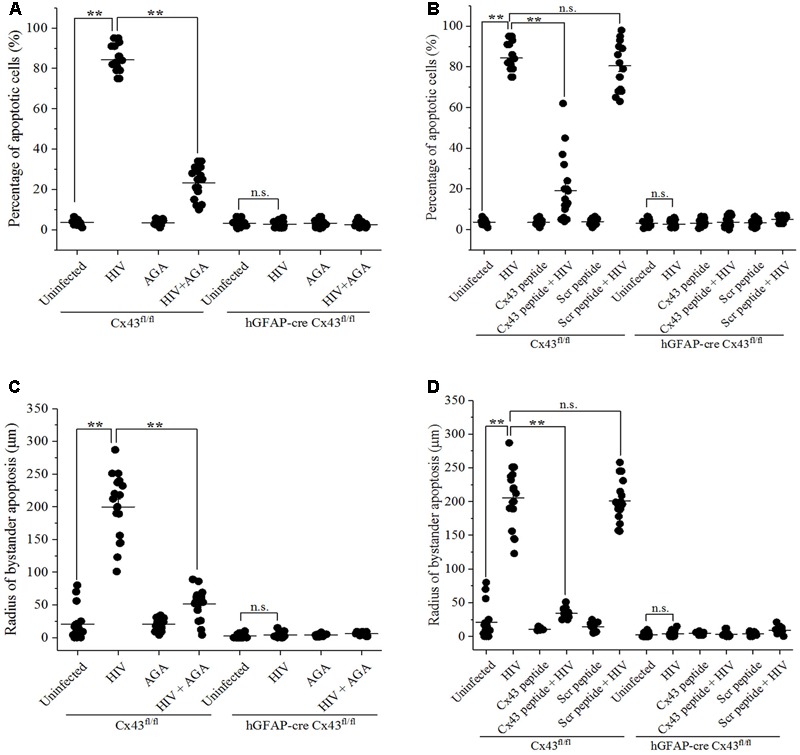
Blocking Cx43 prevents bystander toxicity induced by HIV-infected cells in the mouse brain. Human U87-CD4-CCR5 cells infected with HIV_ADA_ (50 ng/ml) were microinjected into the cortices of Cx43^fl/fl^ as well as hGFAP-cre Cx43^fl/fl^ mice, and simultaneously either GJ blocker, AGA (35 μM), or Cx43 blocking peptide (100 μM) was injected to inhibit GJ- and HC-mediated communication. After 48 h, animals were sacrificed, and brain sections were analyzed for apoptosis by TUNEL assay. **(A,B)** Blocking GJ-mediated communication by AGA **(A)**, and blocking GJ- as well as Cx43 HC-mediated crosstalk by Cx43 blocking peptide **(B)** led to prevention of apoptosis in Cx43^fl/fl^ mice signifying the role of Cx43 in mediating HIV-induced bystander toxicity. However, hGFAP-cre Cx43^fl/fl^ mice had no effect on apoptotic cell death upon injection of either AGA **(A)** or Cx43 blocking peptide **(B)** due to the absence of Cx43 GJs and HCs. **(C,D)** Cx43^fl/fl^ mice where either AGA **(C)** or Cx43 blocking peptide **(D)** was administered along with microinjection of HIV-infected cells, also had significantly smaller radii of bystander apoptosis as compared to Cx43^fl/fl^ mice with active GJ and HC crosstalk, implying the role of Cx43 GJs and HCs in bystander apoptosis. ^∗∗^*p* < 0.005; n.s. – not significant.

As shown in **Figure 3A**, microinjection of HIV-infected U87-CD4-CCR5 cells into cortices of Cx43^fl/fl^ mice led to significant apoptosis around the site of microinjection (84.28 ± 6.38%). However, injection of AGA along with HIV-infected cells prevented apoptosis as these animals exhibited significantly lesser percentage of apoptotic cells (23.30 ± 7.51%), implicating the role of GJs in mediating HIV-induced bystander apoptosis. Similarly, simultaneous injection of Cx43 blocking peptide averted apoptosis (19.22 ± 15.82% apoptotic cells) upon microinjection of HIV-infected cells in the Cx43^fl/fl^ mice (**Figure 3B**). The control animals (Cx43^fl/fl^) where Scr peptide was injected along with HIV-infected cells exhibited 80.56 ± 12.02% apoptotic cells around the site of microinjection, confirming the role of Cx43 GJs and HCs in mediating HIV-induced bystander apoptosis. As expected, microinjecting HIV-infected U87-CD4-CCR5 cells into hGFAP-cre Cx43^fl/fl^ mice cortices either in presence or absence of AGA/Cx43 blocking peptide did not alter the percentage of apoptotic cells due to the lack of Cx43 GJs and HCs (**Figures 3A,B**). These results indicate that Cx43 channels, GJs as well as HCs, are required for bystander killing of uninfected cells surrounding HIV-infected astrocytes.

Similarly, the radius of bystander apoptosis from the site of microinjection of HIV-infected cells was significantly smaller when either AGA (51.61 ± 22.71 μm) (**Figure 3C**) or Cx43 blocking peptide (34.44 ± 6.54 μm) (**Figure 3D**) was injected along with HIV-infected cells in Cx43^fl/fl^ mice as opposed to when the HIV-infected cells were microinjected alone (199.72 ± 49.52 μm). However, in case of hGFAP-cre Cx43^fl/fl^ mice, the addition of neither AGA (**Figure 3C**) nor Cx43 blocking peptide (**Figure 3D**) during microinjection of HIV-infected/uninfected cells had any significant difference in the radius of bystander apoptosis due to the absence of Cx43 channels. These observations reiterate the significance of Cx43 GJs and HCs in mediating bystander apoptosis of uninfected cells during HIV pathogenesis.

### Bystander Apoptosis in Mice Microinjected with HIV-Infected Human Astrocytes Involves Inositol Triphosphate Receptor Activation and Increase in [Ca^2+^]_i_

**FIGURE 4:**
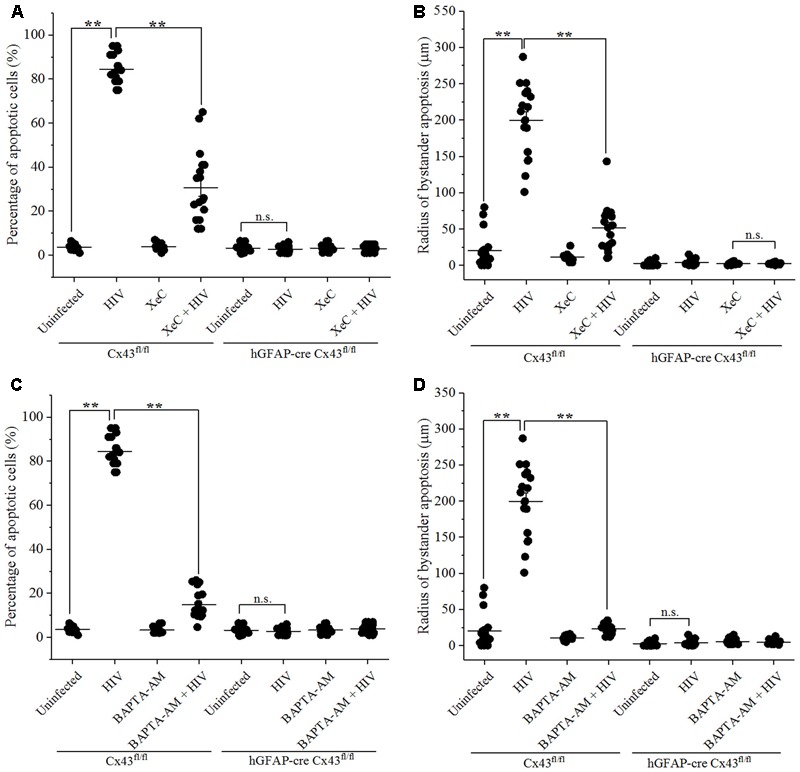
Bystander apoptosis induced by HIV-infected astrocytes microinjected in mice brain involves IP_3_Rs and Ca^2+^ release. Human U87-CD4-CCR5 cells infected with HIV_ADA_ (50 ng/ml) were microinjected into the cortices of Cx43^fl/fl^ as well as hGFAP-cre Cx43^fl/fl^ mice, and simultaneously either IP_3_R blocker, XeC (10 μM), or intracellular Ca^2+^ chelator, BAPTA-AM (100 μM), was administered to assess Ca^2+^ dependency. After 48 h, animals were sacrificed, and the brain sections were analyzed for apoptosis by TUNEL assay. **(A,B)** Bystander apoptosis induced by microinjected HIV-infected cells in Cx43^fl/fl^ mice involved IP_3_R-mediated Ca^2+^ release, since inhibition of IP_3_R activation by XeC prevented bystander toxicity as assessed by percentage of apoptotic cells **(A)** as well as by radius of bystander apoptosis from the site of microinjection **(B)**. **(C,D)** Administration of BAPTA-AM along with microinjection of HIV-infected cells in Cx43^fl/fl^ mice also averted toxicity by regulating [Ca^2+^]_i_ levels leading to lesser percentage of apoptotic cells **(C)** and smaller radius of bystander apoptosis **(D)** as compared to control condition. However, in case of hGFAP-cre Cx43^fl/fl^ mice, use of either XeC **(A,B)** or BAPTA-AM **(C,D)** had no significant effect as these animals did not exhibit bystander apoptosis due to lack of Cx43 GJs and HCs. ^∗∗^*p* < 0.005; n.s. – not significant.

Bystander apoptosis during HIV infection in astrocytes has been reported to involve cytochrome *c*, IP_3_ receptors (IP_3_Rs), and Ca^2+^ release (Eugenin and Berman, 2013). To determine whether bystander apoptosis in mice cortices upon microinjection of HIV-infected cells involved IP_3_Rs and Ca^2+^ signaling, Xestospongin C (XeC), an IP_3_R antagonist, and BAPTA-AM, a cell-permeant Ca^2+^ chelator, were used in the study. At the time of microinjection, either XeC (10 μM) or BAPTA-AM (5 μM) was administered along with HIV-infected/uninfected U87-CD4-CCR5 cells into the cortices of hGFAP-cre Cx43^fl/fl^ and Cx43^fl/fl^ mice. After 48 h, animals were sacrificed and brain sections were analyzed for apoptosis by TUNEL assay. As shown in **Figure 4A**, inhibition of IP_3_R-mediated Ca^2+^ release by XeC resulted in significantly lesser percentage of apoptotic cells (30.55 ± 16.55%) in Cx43^fl/fl^ mice after microinjection of HIV-infected cells as opposed to control conditions (84.28 ± 6.38%). Morover, abolishing increase in intracellular Ca^2+^ levels by addition of BAPTA-AM prevented bystander apoptosis as only 14.91 ± 6.54% cells were apoptotic after microinjection of HIV-infected cells in Cx43^fl/fl^ mice (**Figure 4C**). However, use of either XeC or BAPTA-AM during microinjection of HIV-infected U87-CD4-CCR5 cells into hGFAP-cre Cx43^fl/fl^ mice cortices had no significant difference on the extent of bystander apoptosis as these animals do not express Cx43 GJs and HCs (**Figures 4A,C**). These results suggest that HIV-induced bystander apoptosis involves IP_3_R activation and increase in intracellular Ca^2+^ levels.

Analysis of the radius of bystander apoptosis from the site of microinjection of HIV-infected/uninfected cells revealed similar observations. The use of XeC and BAPTA-AM restricted the presence of apoptotic cells to 51.36 ± 32.34 μm (**Figure 4B**) and 23.28 ± 7.04 μm (**Figure 4D**), respectively, from the site of microinjection in Cx43^fl/fl^ mice, whereas the control mice exhibited much larger radius of bystander apoptosis (199.72 ± 49.52 μm) when HIV-infected astrocytes were microinjected without IP_3_R inhibitor and Ca^2+^ chelator. hGFAP-cre Cx43^fl/fl^ mice did not exhibit any significant differences in terms of radius of bystander apoptosis upon microinjection of HIV-infected cells with either XeC (**Figure 4B**) or BAPTA-AM (**Figure 4D**). These results stregthen our observations that Cx43 GJs and HCs mediate HIV-induced bystander toxicity which involves activation of IP_3_Rs and elevation of intracellular Ca^2+^ levels.

## Discussion

In the current scenario of combined ART, HIV/AIDS has transformed from a life-threatening disease to a chronic disorder that compromises the surviving HIV-infected individuals with accelerated aging, immune reconstitution inflammatory syndrome, ART-associated toxicities, and persistence of the virus in latent hard-to-detect viral reservoirs in anatomical sites including the brain (Johnson and Nath, 2011; Nath and Clements, 2011). Although the prevalence of HIV-associated dementia has declined considerably in the post-ART era, the increasing pervasiveness of the milder forms of neurocognitive impairment still poses a threat to the complete eradication of the virus from the infected individuals (Chan et al., 2016).

Though the extent of HIV infection in the brain is limited to perivascular macrophages, microglia, and a small percentage of astrocytes, the magnitude of HIV neuropathogenesis suggests the involvement of certain host intercellular communication systems that amplify HIV pathology (Malik and Eugenin, 2016). Our laboratory and others have demonstrated that HIV infection targets host intercellular communication systems including GJs, HCs, tunneling nanotubes, and microvesicles/exosomes leading to widespread inflammation (Konadu et al., 2015), bystander apoptosis of uninfected cells (Eugenin et al., 2011), and assist the virus in evading host immune response (Kadiu et al., 2012). Several pathological conditions such as ischemia, stroke and traumatic brain injury, as well as viral and bacterial infections lead to down-regulation of Cx expression and hence GJ-mediated communication (Eugenin et al., 2012). However, during HIV infection, GJ-mediated communication is maintained in astrocytes which enables the diffusion of toxic/apoptotic signals from the few HIV-infected cells to gap-junctionally coupled uninfected cells, thereby amplifying HIV neuropathogenesis (Eugenin and Berman, 2007, 2013; Eugenin et al., 2011).

Our previous studies on HIV-induced bystander toxicity have revealed that HIV-infected astrocytes exhibit mitochondrial dysfunction which leads to an uncontrolled release of cytochrome *c* into the cytoplasm resulting in apoptosis of surrounding uninfected cells (Eugenin and Berman, 2013). We have demonstrated that second messengers, IP_3_ and Ca^2+^, generated in HIV-infected astrocytes as a consequence of mitochondrial dysfunction, diffuse into the surrounding uninfected cells via GJs, leading to their apoptosis since blocking either GJ crosstalk or cytochrome *c*/IP_3_ signaling abolished bystander apoptosis (Eugenin and Berman, 2013). Since the major Cx in astrocytes is Cx43, we wanted to critically examine the role of Cx43 in mediating HIV-induced bystander apoptosis.

HIV encounters dual restriction during infection and replication in the murine cells. First, the interaction between viral envelope protein, gp120, and murine surface receptors, CD4 and CCR5, does not lead to infection in the mouse cells (Browning et al., 1997). Second, HIV transactivating protein (Tat) does not bind to mouse cyclin-T1 and hence cannot activate viral transcription in the murine cells (Garber et al., 1998). Hence, murine models are relevant to study HIV-induced bystander damage in brain cells due to their inability to support HIV infection and replication. To decipher the role of astrocytic Cx43, we used a murine model with an astrocyte-directed deletion of Cx43 gene, hGFAP-cre Cx43^fl/fl^. This mouse model has been extremely useful in studies dealing with astrocyte-specific deletion of Cx43 (Frisch et al., 2003; Theis et al., 2003, 2004; Sridharan et al., 2007), especially because systemic deletion of Cx43 causes early postnatal death (Reaume et al., 1995).

In this study, we have demonstrated that HIV-infected human astrocytoma U87-CD4-CCR5 cells microinjected into the mouse brain, form GJs with resident mouse brain cells, and the apoptotic factors generated in these HIV-infected cells diffuse into uninfected host cells via Cx43 GJs, and lead to their apoptosis. Our results clearly signify the role of Cx43 GJs and HCs in mediating the spread of toxic factors from damaged/pro-apoptotic cells to healthy cells during HIV pathogenesis, as even without supporting HIV infection/replication, mouse brain cells undergo apoptosis owing to the presence of Cx43 protein. The involvement of Cx43 GJs and HCs in bystander apoptosis in the context of HIV is supported by our results from Cx43-deficient hGFAP-cre Cx43^fl/fl^ mouse model, as well as from experiments using GJ blocker and Cx43 blocking peptide in Cx43^fl/fl^ mouse model. Normally, Cx43 is down-regulated in viral infections, but CNS HIV infection is different because HIV uses Cx43 to spread toxicity from few HIV-infected cells to uninfected cells including neurons, astrocytes, and endothelial cells. Thus, Cx43 is used by the non-replicating virus to survive in the host.

Cx43 is the principle Cx in astrocytes, and plays an important role in neuroglia interactions (Malik and Eugenin, 2017). Cx43 knockout animals have been shown to exhibit impaired neuronal plasticity (Han et al., 2014), altered glutamatergic synaptic activity (Chever et al., 2014), and neurodevelopmental defects (Wiencken-Barger et al., 2007). However, limiting GJ-mediated communication during stroke (Wu et al., 2015), ischemia (Contreras et al., 2004), and oxidative stress (Blanc et al., 1998) has been documented to reduce neuronal apoptosis. Moreover, certain GJ blockers have been shown to exhibit neuroprotective as well as cardioprotective effects, and hence partially blocking gap-junctional communication could be favorable in certain pathological events (Herve and Sarrouilhe, 2005; Schulz et al., 2015).

Our study provides significant insights into the role of Cx43 in HIV-induced bystander apoptosis, and is highly relevant since even a low rate of HIV infection in astrocytes represents a significant viral reservoir which could reactivate and repopulate the periphery during ART withdrawal (Li et al., 2016). Since astrocytes form extensive intercellular GJ networks that amplify HIV neuropathogenesis several folds, astrocytes warrant extensive examination to limit the increasing rates of cognitive disease in HIV-infected individuals.

## Author Contributions

SM performed experiments with *in vitro* microinjection, analyzed all data, and wrote the paper. MT designed the study. EAE designed the study, performed all experiments with animal microinjection, and wrote the paper.

## Conflict of Interest Statement

The authors declare that the research was conducted in the absence of any commercial or financial relationships that could be construed as a potential conflict of interest.
