# Supplementary material for: Connexin43 Containing Gap Junction Channels Facilitate HIV Bystander Toxicity: Implications in NeuroHIV
Source: Front Mol Neurosci. 2017 Dec 5;10:404. doi: 10.3389/fnmol.2017.00404 (PMC5723329; doi:10.3389/fnmol.2017.00404)
Supplement: Supplementary file 1 [file Data_Sheet_1.docx]

Supplementary Material

Connexin43 containing gap junctions are required to amplify HIV bystander toxicity: Implications in NeuroAIDS

Shaily Malik, Martin Theis, and Eliseo A. Eugenin

**1. Supplementary data**

**Materials**

Dulbecco’s modified Eagle’s medium (DMEM), FluroBrite DMEM, fetal bovine serum (FBS), penicillin/streptomycin, and trypsin-EDTA were purchased from Thermo Fisher Scientific (MA, USA). U87-CD4-CCR5 cells were procured from NIH AIDS Reagent program (MD, USA). Purified cultures of primary mouse astrocytes, wild-type (WT) as well as Cx43 knock-out (KO), were generously provided by Dr. Eliana Scemes (Albert Einstein College of Medicine, NY, USA). Lucifer yellow (LY) was procured from Sigma (MO, USA). Reusable flexiPERM silicon discs were purchased from Sarstedt (Nümbrecht, Gremany).

**Methods**

**Cell cultures**

To demonstrate GJ-mediated coupling between human and mouse cells, human astrocytoma U87-CD4-CCR5 cells and primary mouse astrocytes were used in the study. U87-CD4-CCR5 cells were maintained in DMEM supplemented with 10% FBS and 1% penicillin/streptomycin. Primary mouse astrocytes, WT as well as Cx43 KO were maintained as described previously (Scemes et al., 1998).

**Lucifer yellow microinjection**

To assess GJ-mediated functional coupling in mouse astrocytes, WT as well as Cx43 KO cells were plated at a density of 2 x 10^5^ cells/dish in 60 mm cell culture dish. After 48 hours, the media was replaced by FluoroBrite DMEM, and the cells were placed in a special Pecon incubation chamber (maintained at 37^o^C, 95% humidity, and supplied with 5% CO_2_) mounted on Zeiss Observer Z1 microscope (Carl Zeiss, Germany). For each cell type, 15 cells were randomly selected, and microinjected with 5% lucifer yellow (LY) in 150 mM LiCl with an Eppendorf FemtoJet Microinjector (Eppendorf, Hamburg, Germany). After 2 minutes, the diffusion of the dye into nearby cells was assessed, and coupling efficiency was calculated. Coupling efficiency refers to the number of cells that allow diffusion of LY from a particular microinjected cell. For determining functional coupling between U87-CD4-CCR5 cells and WT mouse astrocytes, both these cell types were plated at a density of 4 x 10^4^ cells/well, in independent wells of flexiPERM silicon disc inside a 60 mm cell-culture dish. After 24 hours of cell plating, the silicon attachments were removed, and cells were allowed to grow for 5 days to come in physical contact. When the cells were ready, 10-12 U87-CD4-CCR5 cells, which were in close contact with mouse astrocytes, were microinjected with LY. After 2 minutes, the diffusion of the dye into nearby cells was assessed, and coupling efficiency was calculated. For each cell type, 4-5 independent microinjection experiments were performed, and in each experiment 10-15 cells were microinjected.

**Results**

**Functional coupling between U87-CD4-CCR5 cells and mouse astrocytes**

It has been well established that mouse astrocytes form GJs *in vitro* (Basu et al., 2015). To determine functional coupling in mouse astrocytes, we performed LY microinjection in WT as well as in Cx43 KO cells. As shown in Figure S1A, coupling efficiency of WT cells was recorded as 1-20 (from 1 microinjected cell, LY travelled to 20 cells) whereas Cx43 KO cells had a coupling efficiency of 1-0. This reiterates that Cx43 is the major Cx responsible for GJ-mediated communication in mouse astrocytes.

To determine whether human astrocytoma U87-CD4-CCR5 cells and mouse astrocytes can form GJs *in vitro*, both these cell types were cultured in a 60 mm cell-culture dish with a silicon disc (Figure S1B). When the cell density was appropriate for microinjection, 10-12 U87-CD4-CCR5 cells, which were in close contact with WT mouse cells, were microinjected with LY, and diffusion of the dye was analyzed. Figure S1C shows a representative image of a U87-CD4-CCR5 cell microinjected with LY (red arrow), and the diffusion of the dye into a mouse WT astrocyte (white arrow) through GJ channels between the two cells. The formation of GJs between a human cell and a mouse cell *in vitro* strengthens our observations that HIV-infected astrocytes microinjected into the mouse brain form GJs with the host cells, and lead to bystander apoptosis of uninfected host cells.

**Figure legends**

**Figure S1: *In vitro* evidence of coupling between human U87-CD4-CCR5 cells and mouse astrocytes.** Mouse astrocytes, WT as well as Cx43 KO were grown in 60 mm cell-culture dishes, and randomly 15 cells/dish were microinjected with LY. The diffusion of LY was analyzed, and coupling efficiency was determined. **A)** Representative image of a WT astrocyte microinjected with LY (white arrow) and several other astrocytes connected by GJs where the dye diffused (Coupling efficiency: 1-20). In case of Cx43 KO cells, coupling efficiency was determined to be 1-0 as Cx43 serves as the major Cx in mouse astrocytic cells. LY microinjected Cx43 KO astrocyte has been marked with a red arrow. **B-C)** Assessment of the functional coupling between U87-CD4-CCR5 cells (U87) and WT mouse astrocytes was performed by culturing both cell types in independent wells of a silicon attachment having 4 compartments which was placed in a 60 mm cell-culture dish as represented in **(B)**. Randomly 10-12 U87-CD4-CCR5 cells which were in close physical contact with mouse WT astrocytes were microinjected with LY, and diffusion of the dye was analyzed. **C)** Representative image of a U87-CD4-CCR5 cell microinjected with LY (red arrow), and the diffusion of dye into a murine WT astrocytic cell (white arrow), confirming the formation of GJ channels between the two cell types. Scale bar represents 100 μm.

**References**

Basu, R., Banerjee, K., Bose, A., and Das Sarma, J. (2015). Mouse Hepatitis Virus Infection Remodels Connexin43-Mediated Gap Junction Intercellular Communication In Vitro and In Vivo. *J Virol* 90(5)**,** 2586-2599. doi: 10.1128/JVI.02420-15.

Scemes, E., Dermietzel, R., and Spray, D.C. (1998). Calcium waves between astrocytes from Cx43 knockout mice. *Glia* 24(1)**,** 65-73.
