# Supplementary figures and images for: Connexin43 Containing Gap Junction Channels Facilitate HIV Bystander Toxicity: Implications in NeuroHIV
Source: Front Mol Neurosci. 2017 Dec 5;10:404. doi: 10.3389/fnmol.2017.00404 (PMC5723329; doi:10.3389/fnmol.2017.00404)

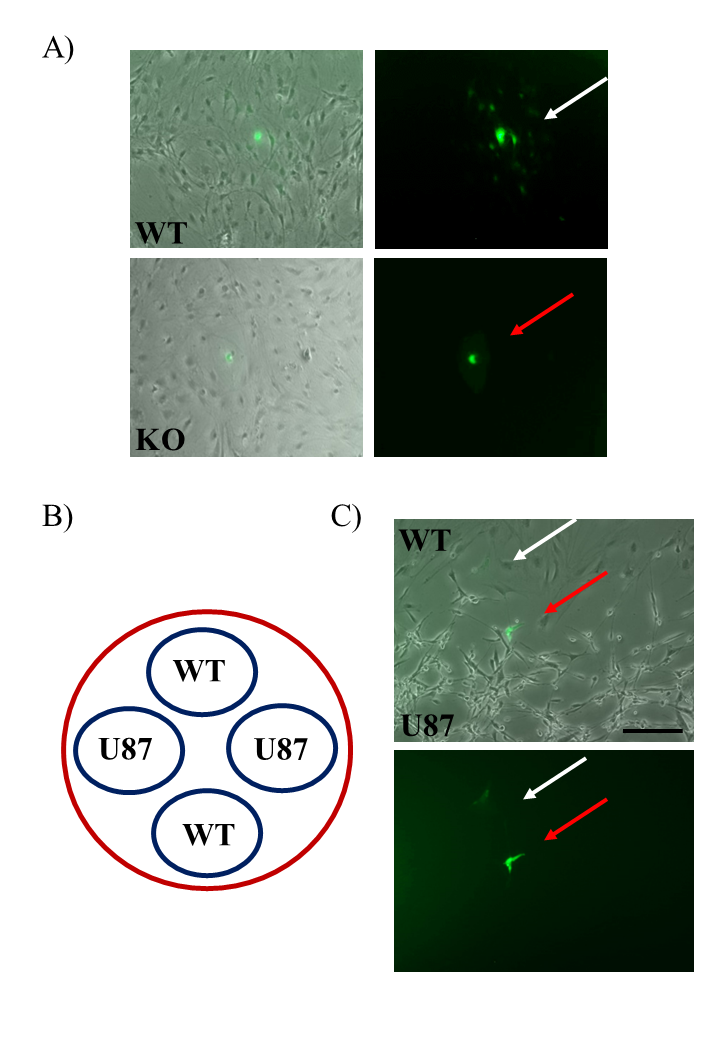

Supplement: Supplementary file 2 [file Image_1.TIF]
